# Sonochemistry of Liquid-Metal Galinstan toward the Synthesis of Two-Dimensional and Multilayered Gallium-Based Metal–Oxide Photonic Semiconductors

**DOI:** 10.3390/mi14061214

**Published:** 2023-06-08

**Authors:** Mohammad Karbalaei Akbari, Nasrin Siraj Lopa, Serge Zhuiykov

**Affiliations:** 1 Department of Solid-State Sciences, Faculty of Science, Ghent University, Krijgslaan 281/S1, B-9000 Ghent, Belgium; nasrin.lopa@ghent.ac.kr (N.S.L.); serge.zhuiykov@ghent.ac.kr (S.Z.); 2 Center for Environmental and Energy Research, Ghent University Global Campus, 119-5 Yeonsu-Gu, Incheon 21985, Republic of Korea

**Keywords:** ultra-thin semiconductors, photonic characteristics, sonochemistry, liquids metals, gallium oxide, selenium

## Abstract

The scientific field of two-dimensional (2D) nanostructures has witnessed tremendous development during the last decade. To date, different synthesis approaches have been developed; therefore, various exceptional properties of this family of advanced materials have been discovered. It has recently been found that the natural surface oxide films of room-temperature liquid metals is an emerging platform for the synthesis of novel types of 2D nanostructures with numerous functional applications. However, most of the developed synthesis techniques for these materials are based on the direct mechanical exfoliation of 2D materials as research targets. This paper reports a facile and functional sonochemical-assisted approach for the synthesis of 2D hybrid and complex multilayered nanostructures with tunable characteristics. In this method, the intense interaction of acoustic waves with microfluidic gallium-based room-temperature liquid galinstan alloy provides the activation energy for synthesis of hybrid 2D nanostructures. The microstructural characterizations reveal the impact of sonochemical synthesis parameters, including the processing time and composition of the ionic synthesis environment, on the growth of Ga_x_O_y_/Se 2D hybrid structures and InGa_x_O_y_/Se multilayered crystalline structures with tunable photonic characteristics. This technique shows promising potential for synthesis of various types of 2D and layered semiconductor nanostructures with tunable photonic characteristics.

## 1. Introduction

Two-dimensional nanostructures and ultra-thin layered nanosheets have recently found numerous applications in the development of functional devices [1]. The properties of these advanced nanostructures have opened up novel opportunities for the design and fabrication of sophisticated engineering systems, where ultra-thin materials play pivotal roles with enhanced functionalities [2]. In accordance with the desired applications, the synthesis methods of ultra-thin nanostructures are continuously developing to provide more flexible methods for the fabrication of novel nanostructured semiconductor-based low-power devices [3]. The gas phase deposition methods, similar to chemical vapor deposition and atomic layer deposition, are technologically advanced and functionally reliable techniques for the development of ultra-thin materials [4,5]. However, the processing of these methods is complicate and requires advanced high-vacuum technologies; therefore, they need large-scale investment. On the contrary, there are other parallel methods that can successfully be employed and developed for practical applications, studies, and evaluation of the properties of novel 2D semiconductor materials [6].

The sonochemical synthesis approaches offer simple routes for the development of nanostructured materials [7,8,9,10,11]. Acoustic ultrasounds are the main factors driving the synthesis of nanostructured materials. Ultrasound and its propagation behavior in the synthesis medium can cause extraordinary situations and generate high-energy sources (13 eV) to spark various kinds of chemical reactions at room temperature [12,13]. The sequential periodic formation, growth, and subsequent collapse of gas bubbles in the sonochemical synthesis process enable not only the fragmentation of layered nanostructures but also the synthesis of novel compounds [14,15]. The initial impact of ultrasonic waves on the materials is related to the mechanical effects of the generated microjets. Mechanical fragmentation is extensively used to delaminate layered structures and diminish their size to their fundamental layers [16]. This technique is commonly used for the synthesis of 2D nanosheets from their layered parent materials. However, in the case of room-temperature liquid metals, sonofragmentation works with different mechanisms. Generally, when high-energy/high-speed microjets are propagating in a reaction medium, strong shear forces enable the drastic mechanical fragmentation of materials, known as the sonofragmentation process [17,18,19]. The liquid metal galinstan cannot be dispersed into small fragments due to its high surface tension. These periodic acoustic waves efficiently break down the room-temperature liquid metal fluids into micro- and then nanosized particles [20,21,22]. This ultrasonic-assisted fragmentation process is the main mechanism for the synthesis of liquid-metal nanoparticles. However, the most interesting phenomena in sonochemistry originates from the interaction of hotspots with the precursors in the reaction environment. High-temperature (5000 k), high-pressure (1000 atm) hotspots (ultrasonic generated bubbles) create exceptional phenomena including plasma formation and nuclear fusion reactions within very small regions inside the synthesis medium [23,24,25]. Such an extraordinary condition in hotspots can induce unusual physical and chemical states and can therefore provide the feasible conditions for the initiation and continuation of chemical reactions, which is followed by the growth of nanostructured materials. When he ionic precursors are also included in the sonochemistry reaction medium, the incorporation, alloying, and synthesis of novel nanostructures with different properties from their parent precursors are expected [26,27,28]. 

Sonochemistry provides two different mechanisms for the synthesis of nanostructures. When sonochemical interactions occur at the core of acoustic bubbles or the so-called hotspot regions, unusual structures and compositions are produced. In fact, the extremely unusual conditions of hotspot cores provide the driving force for the synthesis of nanostructures. For example, the eruption of magma mater into the surrounding environment causes a sharp cooling rate of 10^10^ K/s. Therefore, the synthesized nanostructures experience a quenching process [29]. These combined conditions facilitate the growth of nanostructures in different shapes and compositions. In the second mechanism of sonochemistry, the interactions among the precursors, scattered radicals, and ions occur outside of hotspot regions; and the thermodynamic conditions of the synthesis process are not affected by the extraordinary conditions of hotspots. Therefore, the synthesized materials have properties similar to those of conventional nanostructures. Generally, the parameters of sonochemical synthesis directly affect the structural characteristics and properties of synthesized nanostructures [30,31,32,33]. A typical example is the sonochemical-assisted growth of various types of nanostructured zinc oxides at different synthesis conditions. Previous studies have shown the effect of precursor concentration on the structural characteristics of ZnO [30,31]. With the increase in the concentration of ZnO precursors, the morphology of ZnO changed from 1D nanorods to 2D nanosheets and nanoflakes followed by the multidirectional growth of ZnO-based nanostructures [30,31].

In the present study, we investigated the sonochemistry of liquid-metal galinstan for the synthesis of ultra-thin 2D and multilayered gallium-oxide-based semiconductors. The principal sonochemistry enabled us to investigate the effects of the synthesis conditions on the growth of various types of layered and 2D nanostructures enriched with Se atoms. To meet this aim, liquid-metal galinstan alloy was sonicated in Se containing ionic solutions at different sonochemical synthesis conditions. Subsequently, different types of nanostructures were synthesized. The following nanostructural characterizations and investigation of the photonic characteristics provide valuable information on the synthesis of novel nanomaterials. Here, sonochemical synthesis enabled the growth of crystalline gallium-oxide-based photonic materials enriched with Se nanostructures. This paper describes a functional, feasible, and low-cost synthesis approach, providing great opportunity for the development of novel ultra-thin nanostructures with tunable electronic and photonic properties for numerous functional applications.

## 2. Materials and Methods

In the present study, the sonochemical synthesis technique was employed to develop 2D and multilayered nanostructures. To this aim, 20 mg of galinstan alloy (Indium Corporation, Clinton, NY, USA) was immerged in a glass vial filled with ethyl-alcohol anhydrous (Daejung Chemicals, Republic of Korea). Argon gas was injected into the vial to minimize the presence of oxygen in the synthesis environment. The slurry was probe-sonicated for different durations (1~4 h) at 20 Hz ultrasonic frequency and constant power of 380 W, while the temperature of the slurry was kept at ~20 °C. To synthesize hybrid and alloying 2D and multilayered structures, the synthesis solutions contained ionic Se. To this aim, SeCl_4_ powders were dispersed and dissolved into the reaction medium for the design of the sonochemical synthesis process. The slurries of galinstan liquid-metal particles in anhydrous ethyl-alcohol were probe-sonicated in the ionic solutions at various concentrations (0.1, 0.5, and 1.0 μmol·L^−1^) for different durations (1~4 h). Following the sonication process, the slurry of the synthesized nanostructures was centrifuged to separate the layered nanostructures. In another approach, the floated 2D nanostructures were collected from the surface of the sonication reaction medium. The extracted nanostructures were later used for the structural characterization and evaluation of photonic characteristics.

For material characterization, the 2D and multilayered nanostructures were extracted, dried, and then investigated using various methods. The extracted 2D films were examined without further processing. The micro-Raman studies were performed with a micro-Raman spectrometer (HORIBA Lab Ram ARAMIS, Kyoto, Japan) equipped with λ = 530 and 785 nm lasers. For the investigation of photoluminescence (PL) properties, the PL spectra were collected with a spectrometer equipped with λ = 280 nm laser. X-ray photoelectron spectroscopy (XPS) was later employed for the analysis of the surface composition of the synthesized nanostructures (XPS-Scientific Theta Probe, MA, USA). The absorbance spectra of the samples were measured using a UV–visible spectrometer (Shimadzu, 2600, Kyoto, Japan) to evaluate the bandgap of the materials. The crystallinity was investigated via high-resolution X-ray diffraction (XRD SmartLab, Rigaku Corporation, Tokyo, Japan). A field-emission scanning electron microscope (FE-SEM, JEOL 7800F, Tokyo, Japan) and transmission electron microscope (TEM, JEOL–JEM-F200, Tokyo, Japan) were coupled with energy-dispersive spectrometry (EDS) and selected-area electron diffraction (SAED) equipment to investigate the structural characteristics of the synthesized nanostructures. The high-resolution fast Fourier transform (FFT) patterns of individual regions were collected with the FFT equipment of TEM. For the SEM studies, the extracted nanostructures were drop-casted on Au-coated silicon substrates and dried in a controlled atmosphere at room temperature. For TEM studies, the floated 2D and multilayered nanostructures were dispersed on TEM grids (formvar-/carbon-supported copper grids/400 mesh) and were dried and kept inside of an atmosphere-controlled desiccator before TEM studies.

## 3. Results

### 3.1. Characterization of 2D Ga_2_O_3_ Decorated with Se Nanodomains

**Figure 1:**
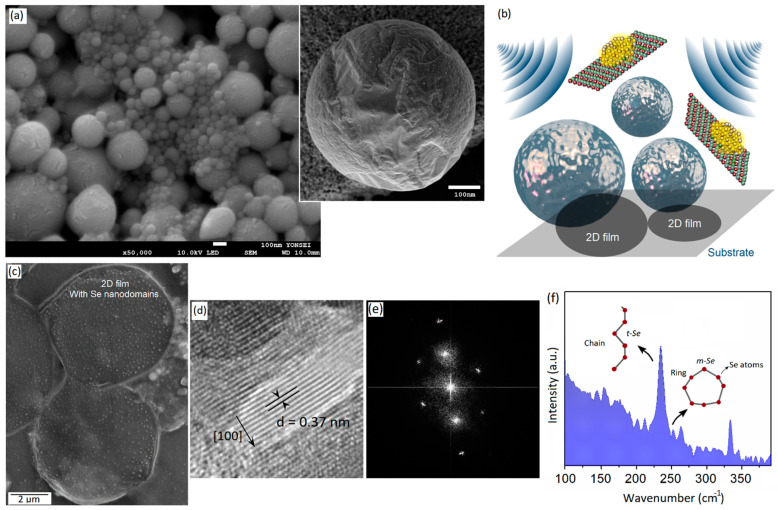
(**a**) SEM image of galinstan NPs synthesized via sonochemical-assisted method accompanied by high-resolution SEM image from surface of a single galinstan NP; (**b**) graphical scheme depicts the mechanism of acoustic-assisted delamination of 2D surface-oxide films from surface of galinstan NPs; (**c**) SEM image of 2D Ga_2_O_3_ films with functionalized nanodomains on their surface (dot-like structures); (**d**) HRTEM image from crystalline Se nanodomains with (**e**) corresponding FFT patterns; (**f**) Raman spectra of 2D Ga_2_O_3_ decorated with Se nanodomains.

Room-temperature liquid metals can feasibly interact with ultrasonic waves. The high-surface tension of LM galinstan effectively suppressed the fragmentation of this room-temperature LM alloy into ultra-fine nanoparticles. The ultrasonic waves provide strong mechanical forces for functional applications. The room-temperature liquid-metal galinstan could be feasibly fragmented into microliquid segments. These fragmented liquid structures sequentially reshape into nanoparticles at the early stage of the sonochemical processing of liquid-metal galinstan in the reaction medium. The initial effect of strong propagation, the outward explosion, and the inward implosion of bubbles during sonication process generated various micro- and nanosized liquid-metal components. Therefore, a wide range of micro- and nanostructured liquid metal particles could be detected in the microstructural studies of the products of the sonication process of galinstan in the liquid medium. The liquid medium had a considerable impact on the shape, morphology, structure, and composition of the sonochemical synthesis products. As an example, the sonication of galinstan in anhydrous ethyl-alcohol resulted in the production of ultra-fine fully spherical galinstan nanoparticles with a uniform particle size distribution (Figure 1a). However, when the sonochemistry reactions occurred in hydrous environments, the interactions of galinstan with the surrounding environment covered a wide range of reactions; therefore, the synthesized products are totally different [34]. The origin of these differences is the complicated reactions between galinstan and the sonochemical synthesis environment. Therefore, the control of the precursor concentration and synthesis conditions of sonochemical reactions tangibly affects the growth and properties of the synthesized nanostructures created by the sonochemistry of galinstan [30,31]. Figure 1a also shows the surface of single galinstan particles synthesized via the sonochemical-assisted functionalization approach. The particle was extracted from the sonication chamber after an hour of sonication (20 Hz and 380 W) of liquid-metal galinstan in anhydrous ethyl-alcohol containing ionic Se solution. The solution contains 0.1 μmol·L^−1^ ionic Se. Our observations showed the growth of crystalline nanodomains on the outer-surface oxide film of the galinstan particles. Generally, the natural-surface oxide film of galinstan can provide a thermodynamically stable substrate for the sonochemical-assisted growth of crystalline metal nanodomains on the surface of galinstan particles [20]. Figure 1d depicts the presence of dot-like structures on the rough 2D surface skin of a galinstan particle. These 2D surface oxide films are ultra-thin and can be separated from the parent galinstan alloy either during a continuous sonication process or even via simple mechanical detachment (Figure 1b,c). Figure 1c depicts the 2D surface oxide film of a galinstan particle that delaminated from the parent metal during the fragmentation of liquid-metal galinstan. These 2D films were primarily composed of Ga_2_O_3_. Gallium oxide is thermodynamically stable structure that can be formed on the outer shell of liquid-metal galinstan [35,36,37]. Uniformly grown nano-dots were observed on the surface of these 2D nanostructures. These nanodomains represent crystalline structures on the surface of 2D Ga_2_O_3_ films. The growth of these nanostructures via sonochemical synthesis represents the second mechanism of sonochemistry; therefore, the developed nanodomains do not mix with galinstan. The following study on the growth of these nanodomains confirmed their crystalline nature. Figure 1d shows an HRTEM image of nanodomains representing the growth of the crystalline plane of Se with a 0.37 nm interlayer spacing aligned in the [100] crystalline direction. We further detected amorphous Se at the early stage of the sonochemical synthesis of galinstan. The following HRTEM studies depicted the corresponding high-resolution fast Fourier transform (FFT) patterns of an individual crystalline Se nanodomain. These patterns belong to the stable α-Se that forms during the sonication process (Figure 1e). The micro-Raman study with a λ = 785 nm near-infrared laser showed the characteristic peaks of either monoclinic Se (m-Se) or trigonal Se (t-Se) in the vicinity of 230~250 cm^−1^. These peaks are attributed to the vibration modes of Se atoms decorated in ring or chain structures (Figure 1f) [38,39]. Notably, changes in the excitation laser source may assist in the detection of Ga–Se structures. As an example, it was observed during our studies that the characteristic vibration modes of Ga–Se bonds could be distinguished when a λ = 785 nm laser was used for Raman spectroscopy, while the same peaks were not recognizable when a λ = 530 nm excitation laser source was employed for characterization. It was found that the λ = 530 nm laser enabled the facile detection of the corresponding Raman peaks of Ga–O bonds. Therefore, we employed a λ = 785 nm laser for the Raman characterization of Se-rich structures. Furthermore, these observations confirmed that functionalized 2D structures can be synthesized via a short-term sonochemical synthesis process. In the early stage of the sonochemistry of galinstan particles in Se-containing ionic solutions, elemental incorporation and alloying process were not observed.

### 3.2. Characterization of 2D Gallium Oxide-Selenide Structures

**Figure 2:**
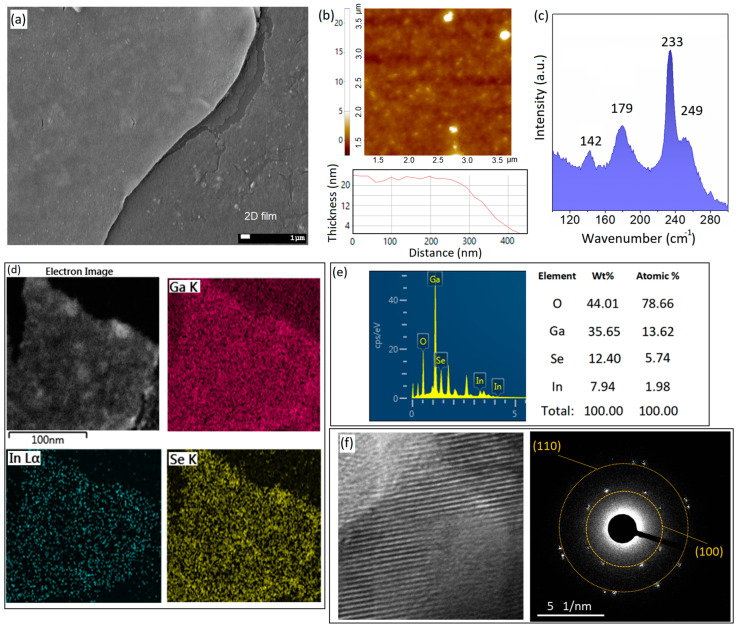
(**a**) SEM image of 2D Ga_x_Se_y_ structures; (**b**) AFM surface morphology and thickness profile of 2D films; (**c**) Raman spectra of 2D Ga_x_Se_y_ structures; (**d**) EDS pattern of surface of 2D films; (**e**) detailed EDS elemental composition results of 2D films; (**f**) HRTEM image and corresponding SAED patterns of 2D films.

When the sonochemical reaction conditions were altered, the byproducts of the synthesis process considerably changed. For example, when the Se concentration in the ionic solution of the sonochemical reaction was increased, layered structures with nanometer-thickness dimensions were observed (Figure 2a). The increase in the precursor concentration produced remarkable changes in important factors such as the viscosity of the sonication medium, vapor pressure, and surface tension. The production efficiency can be affected by the viscosity of the solution, while the vapor pressure can alter the mechanism of sonochemistry from primary to secondary [13]. New conditions can be employed to enable the synthesis of new materials through altered sonochemical reactions. It was previously confirmed that the increase in precursor concentration affects the coalition of the generated bubbles in the reaction environment [13]. Therefore, it can affect the structure and the nature of the nanomaterials synthesized via the sonochemical-assisted technique. It was previously demonstrated that the increase in precursor concentration favors the evolution of structures from nanoparticles into the 1D nanorods; nanosheets; and, later, nanocups, nanoflakes, and other three-dimensional (3D) structures [13,30,31,32]. Here, we also witnessed similar results for the sonochemical synthesis of galinstan liquid metals in ionic Se solution in anhydrous ethyl-alcohol. We observed that the increase in Se content affected the geometries of the synthesized nanostructures. It is understood that crystalline nanodomains are the first products of sonochemical reactions, which develop in the early stages of the sonication process. The 2D surface oxide films of galinstan particles acted as the nucleation sites for the growth of Se crystalline nanodomains (Figure 1). The following increase in Se content in anhydrous ethyl-alcohol to 0.5 μmol·L^−1^ was accompanied by the growth in 2D nanostructures during the 1 h sonication process. Notably, theses 2D nanostructures did not necessarily have the same geometry or characteristics. The details and design of the sonochemical reactions determined the structure of the synthesized 2D films in the present study. In the present study, we observed various types of 2D structures and nanosheets, including ultra-thin 2D films, 2D nanosheets, and ultra-thin multilayered nanostructures. More specifically, the 2D gallium–selenide-rich nanostructures were developed after the sonication of galinstan in Se-containing solutions (0.5 μmol·L^−1^) for 1 h in anhydrous ethyl-alcohol (Figure 2a). These 2D nanostructures were distributed in the sonication medium. The following 48 h resting let the 2D nanostructures be floated on the surface of ionic anhydrous ethyl-alcohol solution. Accordingly, these nanostructures were extracted from the surface of the reaction environment, and then they were studied using different methods. It was observed that the lateral dimensions of these nanostructures varied from a few hundreds of micrometers to a few millimeters. Therefore, these structures could be easily detected (Figure 2a). The AFM studies gave valuable information on the surface characteristics and thickness of the extracted 2D nanostructures. It was observed that the thickness of these nanostructures could vary from 20 nm to 100 nm. Figure 2b shows the surface morphology and corresponding thickness profile of a typical 2D nanostructure. The AFM studies showed the uniform profile of the thickness of the 2D structures. We further investigated the Raman characteristics of the synthesized 2D nanostructures. Figure 2c depicts the typical Raman spectra of the 2D films. The characteristic peaks at 143.3 cm^−1^, 234.1 cm^−1^, and 250.9 cm^−1^ belong to the E_1_, E_2_ and A_1_ vibration modes of t-Se, respectively [38,39,40]. Especially, the A_1_ mode is attributed to the helical chains of Se atoms, confirming the growth of monoclinic rings of Se atoms [38,39,40]. The characterized E bonds also represent the vibration of transverse optical phonons. The individual peak at 179.8 cm^−1^ is the main characteristic of elemental Ga when bonding with Se atoms in a Ga_x_Se_y_ structure [38,39,40]. The following EDS maps of the surface of the 2D nanostructures depicted the presence of Ga, In, Se, and O elements on the surface (Figure 2e). The detailed results of the EDS elemental analysis are presented in Figure 2e. Oxygen and gallium were the most-detected elements on the surface of the 2D films. The EDS analysis of multifaceted Se-rich 2D materials (Figure 2e) showed that the Ga/Se atomic percentage ratio was 2.4%. This displayed that progressive alloying dissociation occurred until the formation of Ga–Se nanostructures. Therefore, we employed TEM equipment to further analyze the composition of the synthesized nanostructures. The high-resolution TEM image from the surface of the 2D films and the collected SAED patterns showed the presence of crystalline phases on the surface of the 2D films (Figure 2f). These observations confirmed the presence of crystalline Se structures on the surface of the 2D films. The characteristic rings of (100) and (110) crystalline planes [20,41,42] were observed in the SAED patterns. These patterns are associated with Se-rich nanostructures, which extensively grew on the outer layer of the 2D films (Figure 2f). The possible explanations include the sonochemical-assisted growth of Se-rich phases. Specifically, the increase in ionic Se in the synthesis environment increased the possibility of the growth in crystalline structures during the sonication stages. Similar observations on the sonochemical-assisted growth of Se nanodomains on nucleation sites were previously reported [20,41].

### 3.3. Characterization of In_x_Ga_y_O-Se Multilayered Structures

We further investigated the effect of the sonication conditions on the development of other ultra-thin nanostructures. It was observed that the increase in the sonication time of the samples containing higher levels of ionic Se affected the structure and morphology of the synthesized materials. The ultrasonication of bimetallic and multi-metallic compounds demonstrated ion-mobility and diffusion phenomena. The effect of sonication time was analogous to the increase in cycles. These increased factors can affect the mass transfer and diffusion processes and, therefore, provide a new condition for the synthesis of new structures [43,44]. To be specific, in the case of the interaction of Se with galinstan, amorphous Se is expected to be formed at an early stage of the sonication process, which later transforms into crystalline structures. 

**Figure 3:**
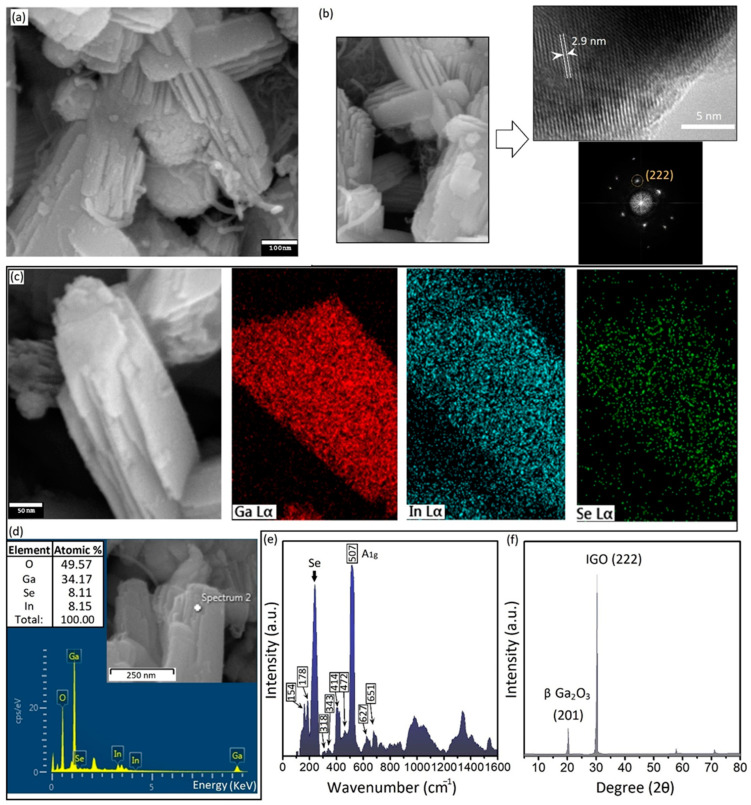
(**a**) SEM image of layered In_x_Ga_y_O/Se structures; (**b**) corresponding TEM image and FFT patterns of these structures; (**c**) EDS pattern of surface of nanostructures; (**d**) detailed EDS elemental composition of these structures; (**e**) Raman spectra; and (**f**) XRD patterns of layered nanostructures.

Ultrasonic waves enable the growth of Se-rich nanostructures under non-thermodynamic conditions [20]. During the sonochemical synthesis, the room-temperature liquid state of galinstan alloy accompanied by the corrosive nature of elemental Se provide feasible condition for the ion-mobility, diffusion, and alloying processes. The first stage of the alloying process starts with the nucleation of Se nanostructures on the surface of galinstan particles, which act as the nucleation sites. The sequential decomposition of SeCl_4_ into SeCl_2_ and later HCl and ionic Se assists with the nucleation of Se on the nucleation sites [27]. HCl alone can remove the natural surface oxide film of galinstan and provide fresh alloys for interactions with ionic elements. During the sonication process, the temperature of the reaction medium was kept lower than or equal to 20 °C. Low-temperature sonochemistry is favorable for the nucleation and growth of bimetallic and intermetallic compounds [34]. In the present study, long-term sonication at a low temperature of 20 °C resulted in the growth of InGaO/Se-based nanostructures. The reduction in ionic Se on the surface of galinstan NPs was accompanied by sonochemical-assisted enhanced interfacial reactive wetting and ionic diffusion. With increasing Se concentration and reaction time, the interfacial reactions increased at the heterointerfaces; therefore, the amalgamation actively continued with the consumption of Se. The long-term sonication process resulted in the formation of hybrid multilayered structures with nanoscale dimensions (Figure 3a). 

These multilayered structures were composed of individual ultra-thin layers with a 20~30 nm thickness. These nanostructures were extensively observed in the byproducts of the sonication process with lateral dimensions within the range of 500 nm to micrometers. To further investigate the material characteristics of these synthesized multilayered nanostructures, TEM studies were performed. Figure 3a shows an SEM image of layered structures after 4 h of sonication of galinstan alloy in a Se-containing ionic solution (1.0 μmol·L^−1^). Layered structures were extensively observed as the byproducts of the sonochemical synthesis process, confirming the effect of the sonication time and precursor concentration on the byproducts of the synthesis process. Figure 3b is the TEM image of the crystalline plane of the layered structures. An interlayer distance of 0.29 nm was measured in different locations on the crystalline structure. 

We further investigated the FFT patterns of these layered structures. The corresponding FFT patterns represented the characteristic crystalline plan of (222) of InGaO (IGO) structures. The interlayer distance of 2.9 is very close to the reported values for the plane distance of the IGO structure [45,46]. The TEM studies produced valuable results, depicting the synthesis of a bimetallic metal–oxide semiconductor, i.e., the IGO nanostructure. This showed that the sonochemical synthesis enabled the formation of binary nanostructures. 

We further conducted the elemental analysis of the layered structures via the EDS equipment of FESEM. Figure 3c specifically depicts the elemental distribution on the surface of a typical layered structure. Ga, In, and Se were present on the EDS map of these nanostructures alongside the oxygen as the fundamental element (Figure 3c). A typical EDS point analysis from the surface of the multilayered nanostructures is presented in Figure 3d. While Ga and O were the main components of these layered structures, the atomic percentage of In and Se were very close to each other (Figure 3d). 

We further analyzed the Raman spectra of synthesized structures. Typical Raman spectra of multilayered structures are presented in Figure 3e. Several peaks were identified in the Raman spectra of these nanostructures. The peaks at 154 cm^−1^ and 178 cm^−1^ were, respectively, attributed to the Se atoms in bonding with other elements [47,48,49]. Especially, the peak at 178 cm^−1^ is characteristic of the binding between elemental Ga and Se atoms in Ga_x_Se_y_ structures [47,48,49]. The sharp and broad peak at vicinity of 200~280 cm^−1^ belongs to the Raman vibration mode of Se atoms. We further characterized the Raman modes of Ga–O bonds at 318, 343, 414, and 472 cm^−1^, which are associated with the deformation of the GaO_6_ octahedral structure. The peak at 507 cm^−1^ is also associated with the A_1g_ vibration mode of In-O bonds. Two other peaks at 627 and 651 cm^−1^ could be attributed to the stretching and bending of the GaO_4_ tetrahedron structure, respectively [50]. The Raman spectra of In_x_Ga_y_O/Se-layered structures showed that the longer sonication process resulted in the growth of novel nanostructures. 

XRD was also employed to further characterize the crystalline structures. Two main peaks were characterized as belonging to the (201) plane of ß-Ga_2_O_3_ and the (222) plane of IGO structures. These characterization results confirmed the synthesis of totally different nanostructures after altering the sonochemistry parameters.

### 3.4. X-ray Photoelectron-Spectroscopy Studies of 2D Structures

**Figure 4:**
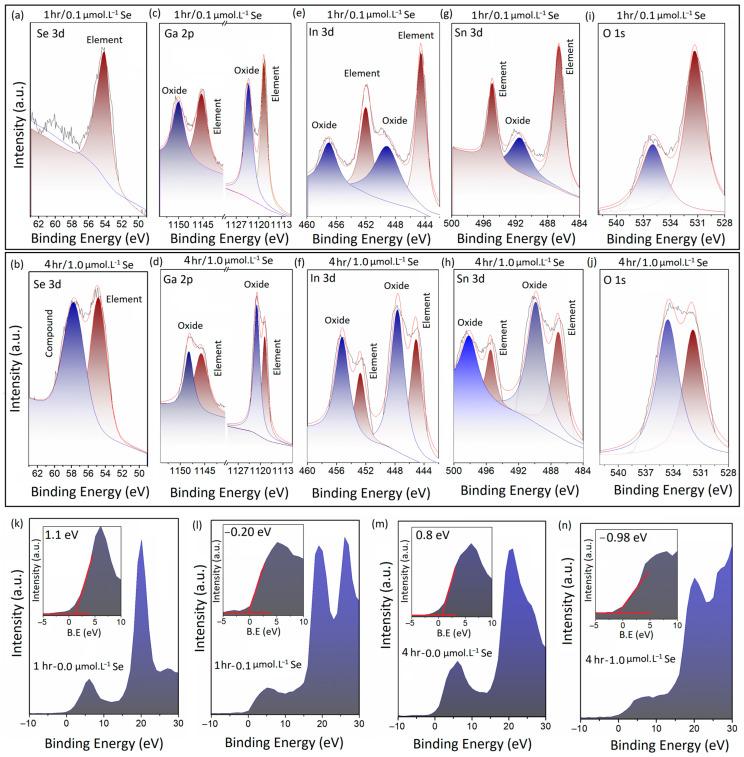
XPS spectra of 2D structures extracted from sonochemical processing of galinstan under different sonication conditions; (**a**,**b**) detailed Se 3d peaks of 2D structures after 1 and 4 h of sonication; (**c**,**d**) detailed Ga 2p peaks of 2D structures after 1 and 4 h of sonication; (**e**,**f**) detailed In 3d peaks of 2D structures after 1 and 4 h of sonication; (**g**,**h**) detailed Sn 3d peaks of 2D structures after 1 and 4 h of sonication; (**i**,**j**) detailed O 1s peaks of 2D structures after 1 and 4 h of sonication. (**k**) VBM of 2D structures extracted after 1 h of sonication in 0.0 μmol·L^−1^ of Se containing solution; (**l**) VBM of 2D structures extracted after 1 h of sonication in 0.1 μmol·L^−1^ of Se containing solution; (**m**) VBM of 2D structures extracted after 4 h of sonication in 0.0 μmol·L^−1^ of Se containing solution; (**n**) VBM of 2D structures extracted after 4 h of sonication in 1.0 μmol·L^−1^ of Se containing solution.

XPS was employed as a characterization technique for the investigation of the oxidation and chemical states of the different components of the 2D and multilayered nanostructures in this study. Figure 4a,c,e,g,i depict the detailed peaks of Se 3d, Ga 2p, In 3d, Sn 3d, and O1s of the extracted 2D structures after 1 h of sonication of galinstan alloy in ionic solution at a concentration of 0.1 μmol·L^−1^ (Se-ethyl alcohol). Figure 4b,d,f,h,j depict the same peaks, related to the multilayered nanostructures after 4 h of sonication in ionic solution at a concentration of 1.0 μmol·L^−1^ (Se-ethyl alcohol). The single Se 3d peak at 54.01 eV in Figure 4a belongs to metallic Se, while the two individual Se 3d peaks (Se 3d_3/2_ and Se 3d_5/2_) in Figure 4b were detected at 54.7 eV and 57.8 eV, which, respectively, belong to the elemental and compound forms of Se atoms. The peak at 57.8 eV could be attributed to the Se atoms bonding with other alloying elements.

The following investigations into the Ga 2p peaks of both samples confirmed the presence of Ga 2p_3/2_ and Ga 2p_5/2_ peaks at 1118.41 eV and 1145.2 eV, respectively. These peaks belong to the metallic form of Ga (Figure 4c). The two other peaks at 1123 eV and 1149 eV are the characteristic XPS peaks of Ga^3+^ of the Ga–O atomic bonds in Ga_2_O_3_ structures (Figure 4c). With the increases in the Se content and sonication time, the intensity of the XPS metallic peaks of Ga decreased, with a small shift toward lower binding energies (Figure 4d). The peaks belonging to the oxidation states of Ga markedly moved to lower binding energies, confirming the considerable alteration in the oxidation states of the Ga atoms in the multilayered structures after the long-term sonication process. Furthermore, it was expected that the element of In was already affected by the sonochemical reactions. The corresponding In 3d peaks of the two groups of samples are presented in Figure 4e,f. The two peaks at 444.4 eV and 452.05 eV in Figure 4e were attributed to the elemental In(0). The two other peaks at 448.9 eV and 456.8 eV, respectively, belong to the In(III) 3d_5/2_ and In(III) 3d_3/2_ of the In_2_O_3_ structure. After the long-term sonochemical reaction, the characteristic peaks of metallic In(0) shifted slightly to higher binding energies (445.0 eV and 452.7 eV), as shown in Figure 4f; the characteristic peaks of In 3d_5/2_ and In 3d_3/2_ of In_2_O_3_ shifted to lower binding energies, i.e., 447.5 eV and 455.2 eV, respectively. More importantly, the intensity of the In 3d peaks of the oxide structures considerably increased compared with that of elemental In(0). This observation was an indication of the changes in the oxidation states of the In atoms after the long-term sonication process. This result suggests the consumption of elemental In(0). 

We further investigated the characteristic Sn 3d peaks of the two samples. In the samples that were sonicated for one hour in a low-concentration Se ionic solution, the peaks of Sn 3d_5/2_ and Sn 3d_3/2_ of metallic Sn were detected at 486.5 eV and 494.0 eV, respectively; while the Sn 3d_5/2_ of the SnO_2_ structure was observed at 491.3 eV. Evidently, the intensity of the corresponding peaks of elemental Sn was considerably higher than that of the oxide. Therefore, we examined the similar peaks in the multilayered sample sonicated for 4 h. Figure 4h depicts two individual peaks of Sn 3d_5/2_ and Sn 3d_3/2_ of metallic Sn at 487.06 eV and 495 eV, respectively. These peaks of metallic Sn also slightly shifted to higher binding energies. Subsequently, two individual peaks of Sn 3d_5/2_ and Sn 3d_3/2_ of the SnO_2_ structure were detected at 489.7 eV and 498.10 eV, respectively. Our observations indicated that the intensity of elemental Sn decreased after the long-term sonication process; meanwhile, the intensity of the corresponding Sn peaks of SnO_2_ considerably increased after the sonication process. Generally, our observations showed that the longer sonication process in higher-concentration Se ionic solutions was accompanied by considerable changes in the oxidation states of the metal elements. In all cases, the Ga 3d, In 3d, and Sn 3d peaks of the metal–oxides experienced tangible increases in their intensities; meanwhile, they experienced an observable shift to lower binding energies. 

We further investigated the O 1s spectra of these two samples. Figure 4i,j demonstrate the O 1s peaks of the synthesized 2D films (1 h sonication) and layered structures (4 h sonication), respectively. The characteristic XPS peak at 531.1 eV could be attributed to the oxygen atoms in the network of the metal–oxide structure (Figure 4i). This peak was also observed at the same location in the XPS spectra of the multilayered structures after 4 h of sonication in 1.0 μmol·L^−1^ Se-containing ionic solution (Figure 4j). A slight shift to higher binding energies of the O 1s peaks was observed after 4 h of sonication. More importantly, two individual peaks were observed in the vicinity of ~536 eV in both samples. These two peaks represent C-O bonds as a characteristic of hydrophilic surfaces and structures [51]. The longer sonication process in the ionic solution containing a higher level of Se ionic was accompanied by the detection of a high-intensity peak in the vicinity of 536 eV (Figure 4j). This interesting observation suggested the effect of Se in the formation of hydrophobic structures and, therefore, the absorption of atmospheric CO to the surface of the synthesized 2D films and multilayered structures. Similar observations were reported in a previous study, where the decoration of a surface-oxide Ga_2_O_3_ film of liquid-gallium-based alloys with Se nanostructures resulted in the enhancement in the CO adsorption on the surface of the 2D films [41]. Moreover, the oxidation of the synthesized Ga–Se-rich nanostructures should be taken into account. 

It is worth mentioning that the sonication process was operated in anhydrous ethyl-alcohol. Moreover, the reaction medium was evacuated from the air and was filled with inert gas. Therefore, the access of the materials to oxygen was restricted during the synthesis process. However, the following material characterization was carried out in the ambient environment; therefore, some level of oxidation was expected. Accordingly, we detected traces of oxygen in the Raman spectra, EDS elemental analysis, and the XPS results of the nanostructures. A previous study also reported the effect of the gradual oxidation of GaSe nanostructures on the material characteristics and photonic properties of GaSe [52]. It was observed that the exposure of GaSe to oxygen was accompanied by the creation of α-Se and Ga_2_Se_3_ [52]. Our XPS studies also confirmed some level of oxidation in the synthesized nanostructures. 

We further examined the effect of the sonochemical synthesis conditions on the valence band maximum (VBM) of the synthesized 2D and multilayered structures. The results indicated that the increase in the Se content in the ionic solution resulted in the decrease in the VBM to lower binding energies. As an example, when the Se content was increased from 0 to 0.1 μmol·L^−1^, the VBM of the 2D films decreased from 1.1 eV to −0.2 eV after one hour of sonication (Figure 4k,l). Similar results were also observed when the Se content increased from 0 to 1.0 μmol·L^−1^ after 4 h of sonication (Figure 4m,n), where the VBM decreased from 0.8 eV to −0.98 eV. More importantly, the sonication time also had an observable impact on the VBM value of synthesized structures. Evidently, a longer sonication time resulted in lower VBM values (Figure 4l,n). These observation suggested the tangible effects of these sonochemical synthesis techniques on the electronic characteristics of the synthesized 2D and layered nanostructures.

### 3.5. The Photonic Characteristics of 2D Structures

**Figure 5:**
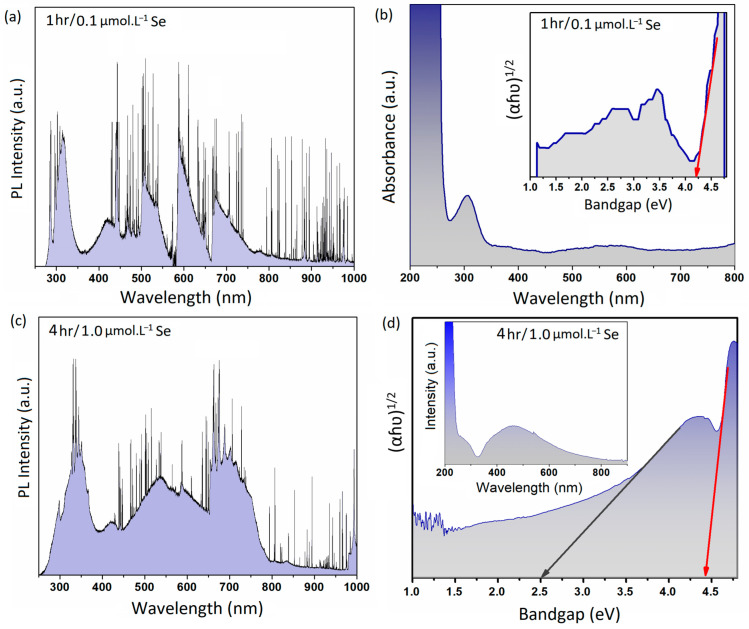
(**a**) PL spectra of 2D structures synthesized after 1 h of sonication of galinstan in 0.1 μmol·L^−1^ and the corresponding (**b**) UV–Vis spectra and bandgap measurements of this sample. (**c**) PL spectra of 2D structures synthesized after 4 h of sonication of galinstan in 1.0 μmol·L^−1^ and the corresponding (**d**) UV–Vis spectra and bandgap measurements of this sample.

To further investigate the photonic characteristics of the 2D structures, we organized a series of measurements. Figure 5a depicts the PL spectra of the 2D structures extracted after an hour of sonication in an ionic solution containing 0.1 μmol·L^−1^ Se. The PL spectra were collected via a λ = 280 nm laser equipped on a Raman machine. Evidently, several peaks were detected after the collection of the PL spectra of the samples. The PL spectra in the UV regions showed a sharp peak at 283 nm and a broad peak in the vicinity of 316 nm. There were also several peaks in the blue region of spectrum. The peaks centered at 419 nm and 463 nm were the main characteristic peaks in the blue regions of spectrum. A distinguished singular peak was also observed in the green region of spectrum, which covers a broad range of 500 to 550 nm. Two more individual peaks in the vicinity of 600 nm and 700 nm were also observed in the red and near-infrared regions of the spectrum (Figure 5a). PL emissions can be associated with electron transition from a donor band to the acceptor and valence bands of 2D structures [53]. The detection of UV emission at λ = 283 nm (4.38 eV) and λ = 316 nm (3.92 eV) could be explained by the detrapping of the electrons and holes due to photoexcitation between the donor and valence bands or the conduction band and valence band [54,55]. 

Electron photoexcitation from the conduction band to valence band is accompanied by electron relaxation, where the electron can freely move from the conduction band to the donor band before the occurrence of radiative recombination in both the UV and green spectra of light. We further characterized the UV–green emission in 2D films synthesized after an hour of sonication in 0.1 μmol·L^−1^ ionic solution. The corresponding peaks with 2.95 eV and 2.67 eV energies could be attributed to the electron/hole recombination between the donor and acceptor bands of the 2D structure [56]. According to our observations, gallium oxide was the main component of the 2D films synthesized with a short sonication time and in low-Se-content solutions. It is suggested that the donor bands are formed by oxygen vacancies and Ga^2+^, while the acceptor bands are shaped by the Ga vacancies and Ga–O-paired vacancies [54]. The broad emission peaks centered in the green, red, and near-infrared regions of the PL spectra of the 2D films (Figure 5a) could have been due to the excitation of the surface plasmon effects of the Se nanodomains. Notably, the red tints were ascribed to the excitation of the surface plasmon resonance of the Se nanostructure [42]. The 2D nanostructures possessed high levels of red and infrared luminesce peaks. Se nanostructures are well known for their photonic, photovoltaic, and semiconducting properties; therefore, these nanostructures can extensively contribute to the performance of 2D Ga_2_O_3_ nanosheets in photonic applications. The Se nanodomains grown on the surface of 2D structures can alter the dangling bands and other surface impurities, which act as the recombination sites for carriers. The presence of metallic Se nanodomains on the surface of 2D structures enhances the density of permanent surface defects and, therefore, influences the PL intensity of these nanostructures [57]. 

We further examined the absorption characteristics of the synthesized 2D structures. The results of UV–Vis measurements of the 2D nanostructures are presented in Figure 5b. These 2D nanostructures were synthesized after an hour of sonication in an ionic solution containing 0.1 μmol·L^−1^ Se. The measured bandgap of 4.23 eV was associated with the 2D gallium oxide structure. 

To further investigate the effect of sonication time and Se concentration on the photonic characteristics of the layered nanostructures, we collected the PL emissions of layered structures synthesized after 4 h of sonication in 1.0 μmol·L^−1^ ionic solution. The PL spectra of these layered structures are presented in Figure 5c. The multilayered structures possessed a high level of mixed luminesce peaks in the green, red, and infrared regions. Similar to the previous samples (Figure 5a), we observed several PL peaks in the UV, blue, green, red, and infra-red regions. In the UV regions, we detected a sharp peak at 298 nm and a large broad peak in the vicinity of 350 nm. This broad peak could be deconvoluted into several other small peaks. The similar peak at 420 nm was related to the blue emission of the layered ultra-thin structures. Furthermore, a broad peak in the vicinity of 550 nm (green emission) overlapped another peak at ~600 nm. We further characterized the broad peak in the near-infrared regions (~700 nm). This peak was previously detected in the PL spectroscopy of the 2D nanostructures. These peaks could be attributed to the excitation and emission peaks of Se nanostructures [41,58]. As was mentioned in the previous section, Se-rich nanostructures experience a high level of surface plasmon excitement in the visible-light regions [58]. 

We also studied the UV–Vis absorbance spectra of the multilayered nanostructures. The extracted UV–Vis results and bandgap measurements of these layered structures are presented in Figure 5d. Two individual bandgap values were detected at 4.45 eV and 2.5 eV. The 2.5 eV bandgap could be attributed the Ga–Se-rich compounds. It was previously shown that the bandgap values of Ga_x_Se_y_ can range from 2.1 to 2.4 eV. Generally, longer sonication time accompanied by higher concentration of Se ions in this sonochemical-synthesis technique resulted in the growth of novel materials with specific photoelectronic characteristics.

## 4. Conclusions

In the present study, we evaluated the ability of sonochemistry to be used for the synthesis of 2D and multilayered metal–oxide semiconductor nanostructures. The sonochemical-assisted synthesis of galinstan alloy in a solution containing Se ions enabled the functional availability for the synthesis of 2D hybrid and multilayered nanostructured semiconductors with different morphologies and photonic characteristics. The material characterization studies revealed that the parameters of the sonochemical synthesis method have tremendous impacts on the final structure and morphology of the synthesized 2D and layered materials. Regarding the synthesis conditions, various types of 2D hybrid and alloying structures were developed. The shorter sonication time resulted in the formation of Ga_x_O_y_/Se 2D hybrid structures; the longer sonication time, in ionic solution with higher ionic concentrations, resulted in the growth of multilayered hybrid crystalline structures with an InGa_x_O_y_/Se chemical composition. The evaluations of the photonic characteristics of these ultra-thin nanostructures demonstrated the suitability of sonochemical-assisted synthesis for the development of 2D or multilayered semiconductor structures with tunable characteristics for photonic applications.

## Data Availability

Available upon request from the authors.
